# Analytical Assessment of Bio- and Toxic Elements Distribution in Pu-erh and Fruit Teas in View of Chemometric Approach

**DOI:** 10.1007/s12011-016-0669-4

**Published:** 2016-04-02

**Authors:** Justyna Brzezicha-Cirocka, Małgorzata Grembecka, Piotr Szefer

**Affiliations:** Department of Food Sciences, Medical University of Gdańsk, Al. Gen. J. Hallera 107, 80-416 Gdańsk, Poland

**Keywords:** Tea, RDA, PTWI, FAAS, Factor analysis

## Abstract

This study concerns application of flame atomic absorption spectrometry (FAAS) in assessment of macro- and microelement and toxic metal levels (Mg, Ca, K, Na, Mn, Cu, Fe, Zn, Cr, Ni, Co, Cd and Pb) in dark (Pu-erh) and fruit tea leaves and their infusions. Phosphorus was also determined in the form of phosphomolybdate by spectrophotometric method. The reliability of the method was checked using three certified reference materials. The results of analysis were in agreement with the certified values, with analytical recovery ranging from 86 to 113 %. Significant correlations (*p* < 0.001) were found between concentrations of P, Zn, K, Ni, Fe, Co, Cr, and Pb in Pu-erh tea, whereas in fruit tea, such interdependences were found between Mg, Fe, P, Ni, and Co. Kruskal-Wallis test results have related differences in Pu-erh tea quality as well as technological processing of fruit tea to their mineral composition. In order to characterize tea elemental content, chemometric techniques such as factor analysis (FA) and cluster analysis (CA) were used. Their application allowed on differentiation of samples in view of the fermentation type, technological processing, and overall quality.

## Introduction

There are approximately 1500 different varieties of tea, which can be grouped by taste and color [1]. The quality of tea depends mainly on the conditions of cultivation, the soil in which it grows, and numerous meteorological conditions. In general, teas are classified into four major types according to the manufacturing process. Among them, there can be listed non-fermented green and white teas, produced by drying and steaming fresh tea leaves. Second group constitutes semi-fermented oolong tea, obtained by a partial fermentation of fresh leaves before drying. Next, fully fermented black and dark teas are produced by a post-harvest fresh leaf fermentation stage before drying and steaming. There is also a post-fermented tea (Pu-erh tea) that undergoes secondary fermentation and oxidation in an open air [1].

Pu-erh refers to the tea plant (*Camellia sinensis* var. *assamica* (L.) O. Kuntze; Theaceae) native to the Upper Mekong River Region of China’s Yunnan Province [2] and contiguous parts of China, Laos, Vietnam, Myanmar, and India [3]. Pu-erh tea is considered to be one of the most aromatic teas that have spicy taste and specific smell. Fine-leaved dark tea, Pu-erh from China, which is particularly well known, goes further fermentation and aging. It can be stored up to 50 years [4] and is used by some consumers as a medicine, tonic, beverage, source of energy, and well-being. Additionally, Pu-erh is valued for nutritive values, aiding digestion, strengthening of the immune system, and obesity prevention [3].

Today, consumers choose not only pure green, black, or dark tea but also that with various types of additives such as fruits. Fruit teas, which are conventionally named as such, should be called a mixture of dried fruits with or without *C. sinensis* leaves. There can be added any fruit, but the most common are apples, lychees, apricots, peaches, berries, and citrus. What is more, there are countries which have a particular favorite kind of fruit added to tea, i.e., dragon fruits are frequently added in South East Asia; in France, black currants; in the USA, pears; and in the Caribbean, mango puree [5].

Tea has numerous beneficial effects on health, such as prevention of low-density lipoprotein oxidation, decreased risk of cardiovascular diseases, and cancers [6, 7]. However, tea contains some undesirable trace elements (heavy metal ions) and organic compounds, such as oxalate. The main sources of heavy metals in tea such as Pb and Cd are fertilizers and local environmental factors [8]. Chen et al. [9] reported that Pb availability increased significantly as the soil pH decreased. Lead enters human body mainly through oral ingestion and absorption through the gut. The absorbed Pb is transferred to soft tissues, including a liver and kidneys, and to bone tissues, where it is accumulated with age. Cadmium’s route of exposure is also through oral ingestion with the diet, and its absorption ranges from 1 to 10 % for adults. The level of Cd in blood is used as an indicator of both recent and cumulative exposures, whereas urinary Cd predominantly reflects cumulative exposure and its concentrations in kidneys [10].

The aim of this study was to assess mineral nutrients and toxic metals levels in 32 kinds of Pu-erh and fruit teas including their infusions. There were also estimated health benefits and hazard associated with this tea consumption in view of permissible dietary limits. Due to the application of chemometric techniques, it was also possible to differentiate quantitatively mineral composition of tea samples and classify them according to the type of fermentation and technological processing.

## Materials and Methods

### Samples

The analyzed tea samples, both in loose form and tea bags, were bought in local markets and tea shops in Poland. The characteristics of dark (Pu-erh) and fruit tea species are summarized in Table 1. Both kinds of tea were analyzed for their content of potassium (K), sodium (Na), calcium (Ca), magnesium (Mg), manganese (Mn), zinc (Zn), copper (Cu), iron (Fe), phosphorus (P), cobalt (Co), nickel (Ni), chromium (Cr), cadmium (Cd), and lead (Pb). In total, 32 types of teas, ca. 300 analytical samples of leaves, and their infusions were analyzed. Blank samples were tested with each series of samples within the same conditions and using the same reagents.

**Table 1:** Characteristics of the analyzed products

No.	Pu-erh tea	Producer	Country/manufacturer’s declaration
Loose form of tea			
1.	Pu-erh grapefruit-orange	Five o’clock	China
2.	Pu-erh Tangela	Five o’clock	China
3.	Pu-erh Royal	Five o’clock	China
4.	Pu-erh warming punch	Five o’clock	China
5.	Pu-erh Yunnan	Five o’clock	China
6.	Pu-erh Superior	Five o’clock	China
7.	Pu-erh	Maraska	China
8.	Pu-erh cranberry	Maraska	China
9.	Pu-erh peach	Maraska	China
10.	Pu-erh strawberry	Maraska	China
11.	Pu-erh cherries in rum	Maraska	China
12.	Pu-erh	Big-Active	China
Tea bags			
13.	Pu-erh	Sir Roger	China
14.	Tea Pu-erh	Carrefour	China
15.	Tea Spa	Irving	–
16.	Pu-erh tea	Teekanne	–
17.	Pu-erh and grapefruit	Vitax	–
	Fruit tea	Producer	Confection
Black tea with fruit			
18.	Intensive Earl Grey and raspberry	Tetley	Bags
19.	Citrus fruits	Lipton	Bags
20.	Forest fruits	Lipton	Bags
21.	Lemon	Lipton	Bags
22.	Intensive Earl Grey and lemon	Tetley	Bags
23.	Lemon and lime	Dilmah	Bags
24.	Raspberry	Dilmah	Bags
25.	Black lemon	Teekanne	Bags
Fruit tea			
26.	Berries	Vitax Family	Bags
27.	Raspberry	Teekanne	Bags
28.	Fresh orange	Teekanne	Bags
29.	Tea garden raspberry	Herbapol	Bags
30.	Tea garden	Herbapol	Bags
31.	Cranberry	Saga	Bags
32.	Cherry	Saga	Bags

### Preparation of Samples

Tea leaves were homogenized using mill IKA® A11 Basic. About 10.0 g (±0.0001 g) of homogenized products portions was weighed and transferred to quartz crucibles. Tea samples were ashed in an electric furnace using a gradient of temperature up to 540 °C. Mineralization procedure was based on the addition of 1.50 mL 36 % HCl (Tracepure®, Merck, Darmstadt, Germany) and 2–3 drops of 63 % HNO_3_ (Tracepure®, Merck, Darmstadt, Germany) to the ashes and evaporation to dryness on a boiling water bath. The dry residue was rewetted with 1.50 mL of 36 % HCl and heated for 1 min, covered with a watch glass. Next, the watch glass was rinsed with ultra-pure water (18 MΩ cm^−1^) from Milli-Q® system (Millipore Corp., Bedford, MA, USA) above the crucible, and the resulting solution was transferred to 25-mL flasks.

In case of infusions, 2.0 g (±0.0001 g) of materials (tea leaves) was weighed and transferred to a 250-mL beaker. Then, tea brew was prepared with 200 mL of water and infused for 5 min under the watch glass cover. Subsequently, the solution was filtered through fluted paper filter and the resulting filtrate was transferred to a quartz crucible. Then, tea infusions were evaporated to dryness on a boiling water bath. The dry residue was ashed in a furnace, following the same procedure as with the dry sample [11].

### Elements Analyses

The concentrations of elements (Mg, Ca, K, Na, Mn, Cu, Fe, Zn, Cr, Ni, Co, Cd, and Pb) were determined by atomic absorption spectrometry with an air-acetylene flame (FAAS) using the deuterium background correction. FAAS conditions are described in Table 2. The analysis was performed using Thermo Scientifics i3000. In case of Na and K, cesium chloride was added (cesium chloride, Merck, Darmstadt, Germany, 0.2 % *w*/*v*), which acts as an ionization buffer that shifts the equilibrium of ion reaction resulting in the free atomic form of the analyzed element. Magnesium and Ca were analyzed with the addition of 0.4 % *w*/*v* lanthanum oxide (lanthanum (III) oxide, Merck, Darmstadt, Germany), which is a correction buffer that connects permanently with the matrix instead of the analyzed element. Phosphorus was determined in the form of phosphomolybdate by spectrophotometric method [12] using UV-VIS spectrophotometer (Spekol 11, Carl Zeiss, Jena, Germany).

**Table 2:** Experimental conditions for element determination by FAAS

Element	Wavelength (nm)	Slit (nm)	Fuel flow (L/min)	Burner width (mm)	Lamp current (mA)	Deuterium background correction
Ca	422.7	0.5	1.0	9.5	6	−
K	766.5	0.5	0.8	6.4	8	−
Mg	285.2	0.5	0.8	6.5	6	+
Na	589.0	0.2	0.8	6.3	8	−
Mn	279.5	0.2	1.0	7.3	12	+
Fe	248.3	0.2	1.0	7.5	15	+
Zn	213.9	0.2	0.8	6.4	10	+
Cu	324.8	0.5	0.8	7.3	5	+
Cr	357.9	0.5	1.2	11.4	5	−
Ni	232.0	0.1	0.9	7.5	5	+
Pb	217.0	0.5	1.0	7.5	10	+
Cd	288.8	0.5	0.8	7.5	8	+
Co	240.7	0.2	0.8	7.3	15	+

### Accuracy of the Analytical Method Used for Quantification

The reliability of the method was checked using three certified reference materials (Tea, NCS ZC73014, Oriental Basma Tobacco Leaves INCT-OBTL-5 and Polish Virginia Tobacco Leaves INCT-PVTL-6). Digestion of these materials was performed with the same decomposition procedure used for tea samples. The recoveries of the studied elements ranged between 86 and 103 %, and the relative standard deviations (RSDs) of the test material were 0.11–14.3 % for Tea, NCS ZC73014. Tobacco INCT-OBTL-5 and INCT-PVTL-6 characterized with recoveries in the range of 87–113 % and RSDs between 0.02 and 10.3 %. All the bioelement and toxic metal measurement results in reference materials are summarized in Table 3.

**Table 3:** Measurements of bioelements and toxic metal concentrations in reference materials, i.e., Tea NCS ZC73014, Oriental Basma Tobacco Leaves INCT-OBTL-5, and Polish Virginia Tobacco Leaves INCT-PVTL-6

Element	Certified concentration (mg/100 g)	Concentration determined (mg/100 g)	RSD (%)	Recovery (%)
Ca^a^ Ca^b^ Ca^c^ Co^a^ Co^b^ Cu^a^ Cu^b^ Cu^c^ Cd^b^ Cd^c^ Cr^a^ Cr^b^ Cr^c^ Mg^a^ Mg^b^ Mg^c^ Mn^a^ Mn^b^ Mn^c^ Zn^a^ Zn^b^ Zn^c^ K^a^ K^b^ K^c^ Na^a^ Na^c^ Pb^a^ Pb^b^ Pb^c^ P^a^ P^b^ P^c^ Ni^a^ Ni^b^ Ni^c^ Fe^a^ Fe^b^ Fe^c^	3260 ± 80 3859 ± 142 2297 ± 78 0.22 ± 0.02 0.10 ± 0.007 18.6 ± 0.7 1.01 ± 0.04 0.51 ± 0.02 0.26 ± 0.01 0.22 ± 0.01 0.45 ± 0.1 0.63^d^ 0.09^d^ 1860 ± 110 853 ± 34 241 ± 9 500 ± 2 18.0 ± 0.6 13.6 ± 0.5 51.0 ± 2 5.24 ± 0.18 4.36 ± 0.1 16,300 ± 700 2271 ± 76 2640 ± 90 90 ± 10 6.24^d^ 1.5 ± 0.2 0.20 ± 0.03 0.10 ± 0.01 4500 ± 0.3 170 ± 12 242 ± 5 3.4 ± 0.3 0.85 ± 0.05 0.15 ± 0.01 242 ± 18 149^d^ 25.8^d^	2818 ± 123 3566 ± 149 2513 ± 13.4 0.21 ± 0.03 0.09 ± 0.005 16.9 ± 0.02 1.00 ± 0.01 0.54 ± 0.01 0.28 ± 0.01 0.21 ± 0.001 0.44 ± 0.02 0.56 ± 0.0001 0.09 ± 0.001 1785 ± 28.8 845 ± 4.96 247 ± 6.01 460 ± 4 20.2 ± 0.34 15.3 ± 0.18 50.9 ± 0.26 5.59 ± 0.24 4.64 ± 0.01 16,713 ± 800 2449 ± 50.3 2692 ± 31.6 88 ± 9 5.50 ± 0.03 1.41 ± 0.03 0.19 ± 0.01 0.09 ± 0.01 4650 ± 0.5 170 ± 0.41 239 ± 0.96 3.2 ± 0.02 0.80 ± 0.078 0.13 ± 0.001 244 ± 14 160 ± 1.01 28 ± 0.30	4.38 4.17 0.5 10.3 5.65 0.12 1.42 2.0 3.48 0.4 4.54 0.02 1.3 1.61 0.59 2.4 0.87 1.67 1.2 0.51 4.31 0.3 4.79 2.06 1.2 10.2 0.5 2.13 5.6 10.3 0.11 0.24 0.4 0.62 9.8 0.6 5.74 0.63 1.1	86 92 109 95 90 91 99 106 105 95 98 89 98 96 99 102 92 112 113 100 107 106 102 108 102 98 88 94 95 89 103 100 99 94 94 87 101 107 109

^a^Tea NCS ZC73014

^b^Oriental Basma Tobacco Leaves INCT-OBTL-5

^c^Polish Virginia Tobacco Leaves INCT-PVTL-6

^d^Information value

### Sensitivity

Limits of detection (LODs) of the method applied for macroelements, Ca, P, Mg, K, and Na, were as follows: 0.02, 0.03, 0.02, 0.04, and 0.02 mg/100 g, respectively. LODs for microelements, Cu, Cr, Zn, Mn, Fe, Ni, and Co, in the analyzed samples were as follows: 0.009, 0.001, 0.02, 0.02, 0.01, 0.002 and 0.003 mg/100 g, respectively. LODs for toxic elements Pb and Cd amounted to 0.004 and 0.003 mg/100 g, respectively. The limits of quantification (LOQs) were defined as LOQ = 3·LOD and were equal to 0.06, 0.09, 0.06, 0.12, and 0.06 mg/100 g for the analyzed macroelements, i.e., Ca, P, Mg, K, and Na, respectively. LOQs of the method for such microelements as Cu, Cr, Zn, Mn, Fe, Ni, and Co amounted to 0.027, 0.003, 0.06, 0.06, 0.03, 0.006, and 0.009 mg/100 g, respectively. LOQs for toxic elements Pb and Cd were equal to 0.012 and 0.009 mg/100 g, respectively. The limit of detection and limit of quantification were established according to Konieczka and Namieśnik [13], i.e. LOD = blank mean + 3SD; LOQ = 3LOD.

In order to validate the analytical protocol, linearity was also evaluated by determining the square correlation coefficients of the calibration curves generated by injections of standard solutions. The data concerning method’s linearity are summarized in Table 4.

**Table 4:** Validation data of the analytical methodology

Element	Linearity		
	Calibration curve range (μg/mL)	Calibration curve	*R* ^2^
Ca K Mg Na P Mn Fe Zn Cu Co Cd Cr Ni Pb	2.00–15.0 0.50–1.50 0.30–0.90 0.50–1.20 0.10–1.20 0.15–5.00 1.00–10.0 0.20–1.00 0.50–4.00 1.00–5.00 0.20–2.00 0.20–2.00 0.50–2.00 0.20–2.00	*y* = 0.06587*x* + 0.0198 *y* = 0.00041*x* + 0.0052 *y* = 0.00131*x* + 0.0236 *y* = 0.00065*x* + 0.0120 *y* = 0.00444*x* + 0.0117 *y* = 0.00015*x* + 0.0067 *y* = 0.05768*x* + 0.0065 *y* = 0.00037*x* + 0.0040 *y* = 0.00011*x* + 0.0049 *y* = 0.00006*x* + 0.0053 *y* = 0.00021*x* + 0.0067 *y* = 0.00006*x* + 0.0013 *y* = 0.00006*x* + 0.0008 *y* = 0.00005*x* + 0.0001	0.999 0.999 0.998 0.998 0.999 0.999 0.998 0.999 0.999 0.999 0.998 0.999 0.999 0.999

### Statistical Analysis

The results were subjected to statistical analysis, i.e., Spearman rank correlation analysis, Kruskal-Wallis test, factor analysis (FA), and cluster analysis (CA), using Statistica 10 (StatSoft®). All selected variables were tested for normality using Shapiro-Wilk test, and normal distribution was not confirmed; thus, non-parametric tests were applied [14]. Data were first subjected to standardization. The data matrix was established using the elements as columns and tea samples as rows. All of the elements were taken into account in the analysis and proved to have a great contribution to sample differentiation. In cluster analysis (CA), the best results were obtained for the Ward’s method using Euclidean distance.

## Results and Discussion

The results concerning the analyzed elements in Pu-erh and fruit teas are listed in Table 5. All the element concentrations (wet weight basis) in tea samples are characterized by arithmetic mean value, the corresponding standard deviation (SD), and ranges.

**Table 5:** Concentration of bioelements and toxic metals in Pu-erh (loose/bags) and fruit tea (bags) samples in milligrams/100 g w.w. ($\overset{-}{x}$±SD, range) and an average percentage of leaching (%)

Elements	Pu-erh loose form (China)	Pu-erh tea bags (marketed)	Fruit tea (tea bags)	Black tea with fruit (tea bags)
*n*	12 × 6	5 × 6	7 × 6	8 × 6
Ca	872 ± 352 (325–1437) 15.9 ± 19.7 %	657 ± 144 (457–845) 9.90 ± 6.71 %	727 ± 154 (531–978) 42.9 ± 11.4 %	632 ± 318 (120–1020) 25.3 ± 19.1 %
K	2653 ± 186 (2386–3085) 56.9 ± 19.7 %	2457 ± 454 (1742–3141) 46.7 ± 19.1 %	1113 ± 555 (410–2167) 76.5 ± 8.86 %	1275 ± 137 (1008–1467) 68.4 ± 17.5 %
Mg	322 ± 49.2 (271–397) 17.8 ± 7.35 %	307 ± 45.0 (242–379) 25.4 ± 3.88 %	193 ± 88.8 (90.1–336) 55.0 ± 20.2 %	118 ± 26.0 (79.6–151) 56.6 ± 4.69 %
Na	16.2 ± 5.40 (10.0–27.6) 40.6 ± 13.1 %	27.1 ± 13.1 (15.0–50.6) 45.3 ± 18.9 %	11.8 ± 4.73 (4.11–18.4) 67.7 ± 24.8 %	8.32 ± 5.08 (3.89–17.6) 56.9 ± 23.2 %
P	1421 ± 180 (1261–1940) 26.6 ± 2.21 %	1356 ± 208 (948–1526) 23.6 ± 1.10 %	519 ± 134 (334–781) 15.0 ± 3.50 %	881 ± 64.0 (778–979) 14.5 ± 1.67 %
Mn	77.2 ± 12.0 (47.3–94.2) 15.7 ± 3.42 %	76.6 ± 10.4 (56.7–84.9) 15.8 ± 3.04 %	21.7 ± 5.82 (13.0–30.0) 56.2 ± 8.28 %	76.2 ± 27.6 (34.0–115) 19.5 ± 6.94 %
Fe	32.2 ± 5.81 (16.8–41.6) 2.37 ± 0.71 %	68.3 ± 10.2 (50.9–78.3) 1.65 ± 0.36 %	33.7 ± 10.7 (18.2–50.2) 2.64 ± 1.07 %	21.8 ± 6.93 (14.0–35.4) 0.91 ± 0.55 %
Zn	3.84 ± 0.42 (3.38–4.73) 29.1 ± 12.6 %	3.77 ± 0.68 (2.45–4.43) 32.2 ± 7.73 %	2.04 ± 0.52 (1.47–3.00) 46.0 ± 17.0 %	2.6 ± 0.53 (1.95–3.60) 39.2 ± 13.9 %
Cu	1.93 ± 0.17 (1.66–2.21) 5.15 ± 3.98 %	1.94 ± 0.38 (1.27–2.44) 11.6 ± 2.00 %	0.47 ± 0.10 (0.38–0.65) 31.9 ± 7.95 %	1.1 ± 0.81 (0.14–2.72) 43.1 ± 27.3 %
Co	0.02 ± 0.005 (0.01–0.03) 3.76 ± 1.47 %	0.03 ± 0.006 (0.02–0.04) 3.81 ± 0.35 %	0.02 ± 0.01 (0.01–0.04) 3.62 ± 2.69 %	0.04 ± 0.02 (0.02–0.06) 2.12 ± 1.31 %
Cd	0.02 ± 0.001 (0.02–0.02) 4.67 ± 2.44 %	0.02 ± 0.001 (0.02–0.02) 2.80 ± 1.40 %	0.02 ± 0.003 (0.02–0.03) 8.24 ± 4.29 %	0.02 ± 0.001 (0.02–0.02) 5.18 ± 1.73 %
Cr	0.15 ± 0.03 (0.09–0.19) 45.6 ± 15.4 %	0.33 ± 0.04 (0.28–0.38) 20.0 ± 2.78 %	0.24 ± 0.09 (0.09–0.41) 40.4 ± 30.2 %	0.22 ± 0.13 (0.08–0.48) 29.2 ± 7.70 %
Ni	0.60 ± 0.06 (0.49–0.71) 34.3 ± 17.1 %	0.52 ± 0.08 (0.36–0.60) 45.3 ± 2.86 %	0.19 ± 0.04 (0.12–0.25) 42.6 ± 14.2 %	0.46 ± 0.09 (0.35–0.57) 66.8 ± 11.7 %
Pb	0.04 ± 0.02 (<LOD–0.08) 37.2 ± 22.9 %	0.09 ± 0.03 (0.04–0.13) 15.8 ± 3.28 %	0.01 ± 0.002 (<LOD–0.03) 43.1 ± 23.0 %	0.01 ± 0.008 (<LOD–0.02) 43.8 ± 29.5 %

*n* number of samples multiplied by number of analytical subsamples

LOD for Pb = 0.004 mg/100 g

### Pu-erh Tea

Pu-erh tea was characterized by high levels of K, P, Ca, and Mg (2653, 1421, 872, and 322 mg/100 g, respectively), and these elements were more abundant in leaf tea than in tea bags. The content of Na in marketed Pu-erh tea bags (27.1 mg/100 g) was higher than in a loose form (16.2 mg/100 g), which is in agreement with results published by McKenzie et al. [15]. Similar results to our study were reported by Malik et al. [16] for such elements as Ca, K, and Mg amounting to 716, 2176, and 297 mg/100 g, respectively. The highest percentage of leaching was recorded for K and Na (46.7–56.9 and 40.6–45.3 %, respectively) and the lowest for Ca (9.90–15.9 %). Among microelements, the highest concentration was determined for Mn (77.2 mg/100 g) in marketed tea bags and the lowest for Co (0.03 mg/100 g) in loose form of Pu-erh tea. In general, higher levels of Fe, Co, Cr, and Pb were noted for tea bags than loose tea. Seenivasan et al. [17, 18] suggested that high Cr content in tea can be explained by the contamination of tea primarily from soil and CTC rollers during tea production. The results obtained for Cu in both forms of Pu-erh tea (1.93 mg/100 g) are similar to those reported by Cao et al. [19] (2.10 mg/100 g) and Malik et al. [16] (2.10 mg/100 g). Pu-erh tea in loose form contained more Ni (0.60 mg/100 g) as compared with black tea with fruit (0.46 mg/100 g). Zinc and Fe levels (3.80 and 32.0 mg/100 g, respectively) were similar to those obtained by McKenzie et al. [15] (3.40 and 38.0 mg/100 g, respectively). The lowest percentages of leaching in tea bags were noted for Fe, Cd, Co, Ca, and Cu (1.65, 2.80, 3.81, 9.90, and 11.6 %, respectively). Karak and Bhagat [20] reported comparable results of leaching Cr (16.0 %) to our study (Cr 20.0 %). Lead and Cd levels in samples of Pu-erh tea amounted to 0.06 mg and 0.02 mg/100 g, respectively. Comparable results for Cd were reported by Lv et al. [21] (0.02 mg/100 g).

### Fruit Tea

The highest K and Ca levels (1113 and 727 mg/100 g, respectively) were determined in teas consisting only from fruits. In case of black teas with fruit additions, the highest values among macroelements were recorded for K and P (1275 and 881 mg/100 g). Similar K values (1135 mg/100 g) were reported by McKenzie et al. [15]. Black tea with fruit analyzed in our study was characterized by higher values of P (881 mg/100 g) as compared with results obtained by McKenzie et al. [15] for black teas (286 mg/100 g). Bioelements that characterized with the highest percentages of leaching were K (68.4–76.5 %) and Na (56.6–67.7 %). Among microelements, the lowest level was determined for Co (0.03 mg/100 g), which is comparable to results obtained by Pękal et al. [22] (0.02 mg/100 g). Among microelements, the highest levels were obtained for Mn in black tea with fruits (76.2 mg/100 g). Similar values for Mn (64.0 mg/100 g) were reported by Mehra and Baker [23]. Jonah and Williams [24] suggested that tea foliage accumulates Mn in appreciable quantities with values as high as 100 mg/100 g in tea leaf grown in acid soils, which is in agreement with our study (Pu-erh tea and black tea with fruit). Black teas with fruit additions contained lower levels of Fe (21.8 mg/100 g) than fruit teas. This result is in agreement with values published by Ashraf et al. [25] and Zeyuan et al. [7]. Based on our results, it was also concluded that fruit teas contained more Pb than black teas with fruits, whereas Cd in both kinds of tea was on the same level (0.02 mg/100 g). Jin et al. [26] reported that black tea leaves contained Pb in the range of 0.02 and 0.04 mg/100 g. The highest percentage of leaching was recorded for Ni and ranged from 42.6 to 66.8 %.

Differences in element concentration in infusions might be explained by chelation of these elements with tannic acid and tannins which exudate during the boiling of tea. Dambiec et al. [27] and Wróbel et al. [28] reported that lower content of tannins in tea results in better extraction of elements to the infusion.

### Correlation Analysis

Correlation analysis was performed using the nonparametric Spearman rank test for the analyzed elements at three levels of significance (*p* < 0.05, *p* < 0.01, *p* < 0.001). The most significant positive relationships (*p* < 0.001) in Pu-erh tea were noted for P-Zn, P-Ni, K-Ni, K-Zn, Fe-Co, Fe-Cr, Fe-Pb, Co-Cr, and Cr-Pb. In case of fruit teas, such significant positive interdependences were recorded for Mg-Fe, P-Ni, and Co-Ni.

### Kruskal-Wallis Test

Kruskal-Wallis test was performed for Pu-erh and fruit teas. It was found that the type of tea confection (loose or tea bag) is linked to concentrations of such elements as Fe (*H* = 10.000; *p* = 0.0016), Co (*H* = 7.511; *p* = 0.006), Cr (*H* = 10.000; *p* = 0.002), Pb (*H* = 6.944; *p* = 0.008), and Na (*H* = 4.444; *p* = 0.035). Strong interdependences were also observed in samples of fruit teas and black teas with fruit additions for such elements as P (*H* = 9.422; *p* = 0.002), Mn (*H* = 10.500; *p* = 0.001), Fe (*H* = 4.339; *p* = 0.037), Ni (*H* = 10.500; *p* = 0.001), and Cd (*H* = 7.085; *p* = 0.008). The null hypothesis of Kruskal-Wallis test was rejected, so a post hoc test for the multiple comparisons was applied, which allowed on differences’ identification. Several elements have been identified as being dependent on the type of tea and its sort of confection. However, such interdependences were not found in case of Ca. What is interesting, it was found that Cr is strongly correlated only with the sort of confection, which can imply the influence of technological processing. All the results are presented in the Table 6. The results of post hoc analysis confirm the outcome of factor analysis.

**Table 6:** Results of the post hoc test conducted for the analyzed data matrix

	Pu-erh loose tea	Pu-erh bags	Fruit tea	Black tea with fruit additions
Pu-erh loose tea	–	Cr	K, P, Mn, Zn, Cu, Ni	K, Mg, P, Zn, Co, Cd, Pb
Pu-erh bags	Cr	–	K, P, Mn, Zn, Cu, Pb	Mg, Na, P, Fe, Cd, Pb

There are only given elements for which *p* < 0.05

Kruskal-Wallis test conducted by McKenzie et al. [15] revealed that there are strong interdependences in tea samples for elements: Mn, Zn, Fe, P, and Na in view of geographical origin. Similarly, Moreda-Pineiro et al. [29] found statistically significant differences for Fe, Mn, and Pb for tea samples in view of geographical origin.

### Factor Analysis

**Fig. 1:**
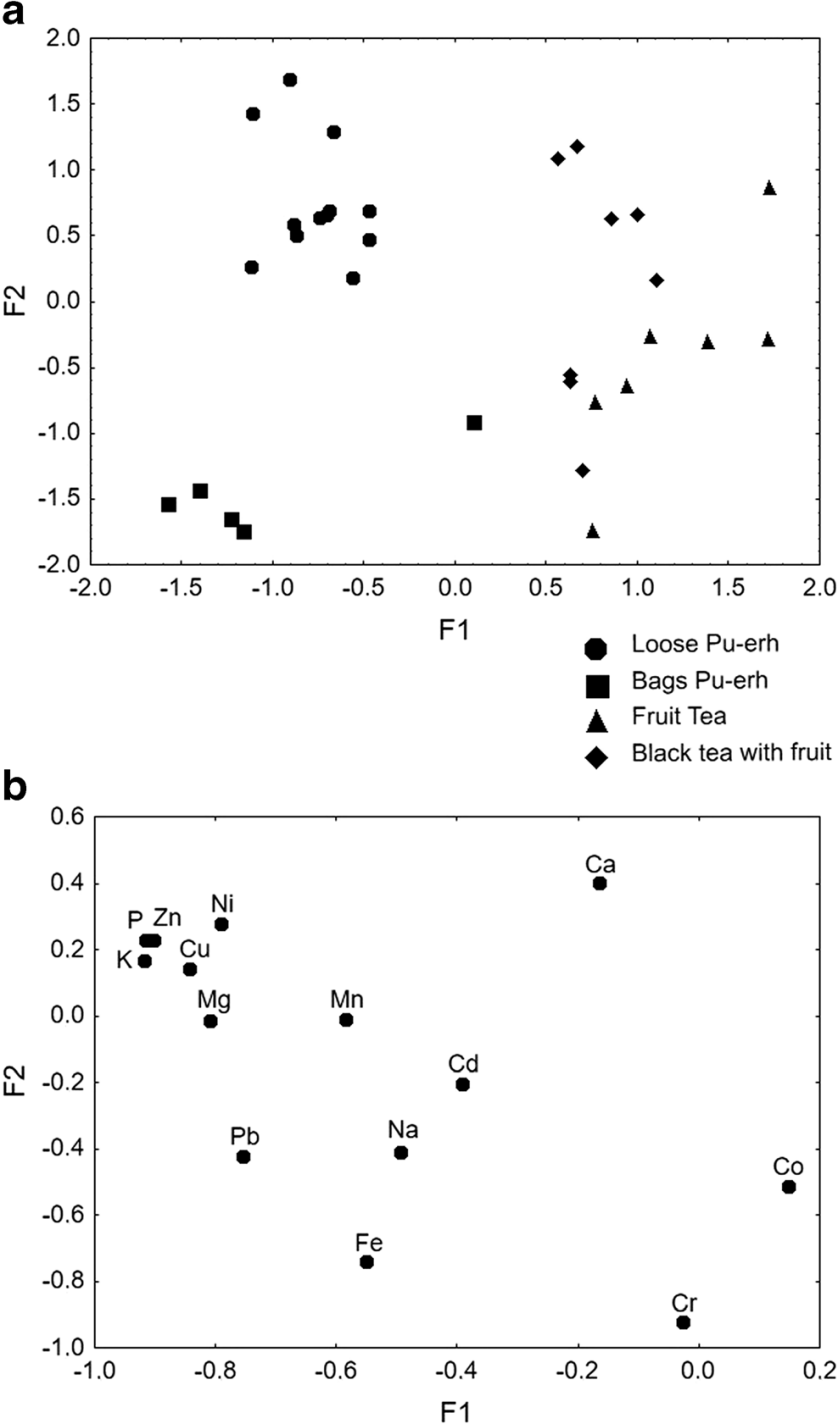
**a** Scatterplot of object samples of two discriminant functions of the all analyzed tea samples. **b** Scatterplot of loadings for 14 elements in all the analyzed tea samples

Factor analysis (FA), which was performed on raw data sets of Pu-erh and fruit teas, revealed two factors (F1 and F2) that cumulatively explain 61.36 % of the total variance (F1 = 43.93 % and F2 = 17.42 %). Eigenvalues amounted to 8.59 for F1 and 6.15 for F2. It can be observed on Fig. 1a that samples of Pu-erh tea (lower values of F1) are clearly distinguished from fruit teas characterized by higher values of F1 (Fig. 1a). The scatterplot of loadings was drawn for F1–F2 in order to identify elements responsible for the grouping of objects (Fig. 1b). Factor 1 reveals a high (negative) correlation to P, K, Zn, Cu, Ni, Mg and Pb, Mn, Fe, and Na, which characterize Pu-erh teas that contain higher levels of these elements (Table 5) than fruit teas and black teas with fruit additions. That is why, Pu-erh teas appeared at more negative values of F1 than fruity ones (Fig. 1a). Additionally, F2 explains the sample differentiation, cumulatively with F1, as there is clear diversification in view of the type of confection among Pu-erh tea samples (lower F1 values). Higher values of F2 and lower of F1, which are identified by P, Zn, Ni, K, Cu, Mg, and Mn, can be associated with loose form of Pu-erh samples, whereas F2 lower values (lower F1 values) correspond to Pu-erh tea bags, characterized by Fe and Cr (Fig. 1a, b). Chromium and Fe are strongly negatively correlated (>0.7), and their concentrations are significantly varied in loose and bags teas. Although Pb, Na, and Co characterize with negative F2 loadings (>0.4), there are no significant differences in their levels in the analyzed teas.

Simultaneously, F2 is helpful in obtaining extra information concerning fruit and black teas with fruit addition samples, which are described by higher values of F1. Although, these samples partially overlap, but there is also observed diversification according to their type, i.e., black tea with fruit additions are characterized by Ca, and higher values of F1 and F2 (Fig. 1a, b). Although Cr and Fe are strongly negatively correlated with F2, there are no significant differences in their concentrations in both groups of fruit teas; thus, they are not differentiating these samples (Table 6).

Based on the conducted analysis, it can be concluded that F1 is a factor responsible for differentiation of samples in view of the tea type (Pu-erh and fruit teas). However, factor 2 provides additional to F1 information concerning the analyzed samples, and both of them cumulatively are responsible for the diversification within the group of fruit tea, i.e., pure fruit tea from black tea with fruit additions as well as Pu-erh tea in view of its confection (loose form–tea bags) which is usually associated with its quality.

Most of the authors differentiated teas in view of their type and geographical origin [15, 29–31]. In our study, tea diversification was achieved according to its kind and type of confection.

### Cluster Analysis

**Fig. 2:**
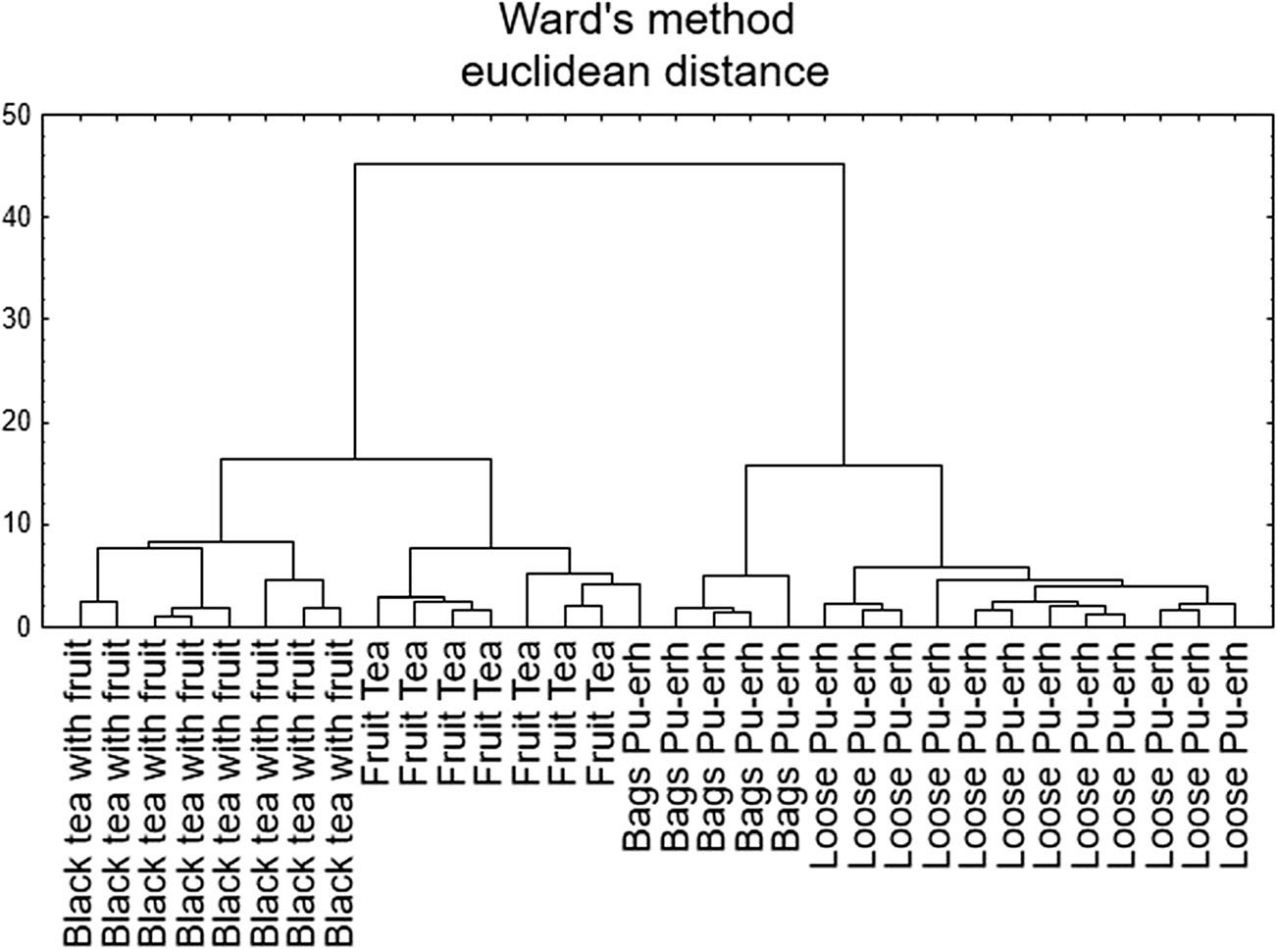
Hierarchical dendrogram for the analyzed tea samples as objects

The cluster analysis (CA) was performed using Ward method and Euclidean distance on data concerning Pu-erh and fruit tea samples. Figure 2 presents the outcome of the conducted analysis, i.e., the dendrogram which is built of four main clusters containing samples grouped according to the type of confection (loose, tea bag) and kind of tea (Pu-erh tea, fruit tea, and black tea with fruits). The first cluster contains samples of black tea with fruit additions which characterized with the lowest Ca levels among all analyzed samples. Next to them, there can be distinguished cluster with fruit tea samples that were differentiated in this analysis by Mg and K, which are metals of natural soil origin. It is understandable as fruits are usually reckoned as sources of Mg and K [32]. However, in case of Pu-erh tea samples, differentiation was based on metals of anthropogenic origin. Chromium, Co, and Fe proved to be good descriptors of type of tea confection, which can be observed on Fig. 2, where the third and the fourth clusters correspond to Pu-erh tea bags and Pu-erh loose tea, respectively. In case of Cr, there can be observed higher levels of this metal in tea bags mainly due to the applied technological processing, i.e., CTC [17, 18].

As can be observed, CA made it possible to differentiate tea samples in view of the tea quality and its type with respect to the chemical composition of the analyzed samples.

### Recommended Dietary Intake

The recommended daily intake (RDA) was calculated for Pu-erh and fruit tea infusions according to the latest available Polish [33] and American recommendations [34]. RDAs for macroelements such as Ca, K, Na, Mg, and P, through consumption of 200 mL of Pu-erh and fruit teas, were realized from 0.007 to 1.00 % (Table 7). Similar percentages of the RDA realization can be observed in the case of Zn and Fe. Chromium, Ni, Cu, and Co were under the limit of detection; thus, there could not have been estimated the percentage of recommended daily intake realization for these elements (Cr < 0.001 mg/100 g, Ni < 0.002 mg/100 g, Cu < 0.009 mg/100 g, and Co < 0.003 mg/100 g). The highest RDA realization through consumption of one cup daily was recorded for Mn (10.0–15.0 %), which constitutes significant values, but its bioavailability to human body should be also taken into account. According to Powell et al. [35], Mn in simulated intestinal conditions was bioavailable in 40.0 %. It means that only about 50.0 % of Mn determined in the studied Pu-erh and fruit tea samples was bioavailable. Mossion et al. [36] also suggested that water composition plays an important role in chemical extraction of elements from tea leaves and strongly determines tea infusion composition. Additionally, it is a well-known fact that there are many anti-nutritive substances in tea such as oxalate or tannins which can absorb many metals in solution. According to the World Health Organization (WHO), the provisional tolerable weekly intake (PTWI) for Cd should not exceed 7 μg/kg, for a person weighing 70 kg. In case of Pb, the 73rd report of the Joint FAO/WHO Expert Committee on Food Additives [37] announced that its PTWI, which previously amounted to 25 μg/kg of body weight for 70 kg adult person, could no longer be considered health protective and was withdrawn. Moreover, the committee stated that based on the available data, it was not possible to establish a new PTWI that would be health protective [37, 38]. However, as levels of these two elements in all tea infusions were under the detection limits of the method applied (Pb < 0.004 mg/100 g and Cd < 0.003 mg/100 g), therefore, it was concluded that there is no health hazard associated with consumption of Pu-erh and fruit teas. Similar results were obtained by [39].

**Table 7:** Realization of the recommended dietary intake through consumption of one cup (200 mL) of Pu-erh (loose/bags) and fruit tea/black tea with fruit

Element	Recommended daily allowance (RDA) (mg/day/person)		Pu-erh (loose/bags) Average content (mg/200 mL)	Fruit tea/black tea with fruit Average content (mg/200 mL)	Pu-erh (loose/bags) Realization of RDA through consumption of 200 mL of infusion (%)		Fruit tea/black tea with fruit Realization of RDA through consumption of 200 mL of infusion (%)	
	Male	Female			Male	Female	Male	Female
	(31–50 years)	(31–50 years)			(31–50 years)	(31–50 years)	(31–50 years)	(31–50 years)
Ca	1000	1000	1.60 ± 1.11	4.00 ± 2.66	0.16	0.16	0.40	0.40
			0.19–4.70	0.09–7.64				
K	4700	4700	28.2 ± 11.8	17.3 ± 6.79	0.60	0.60	0.37	0.37
			13.3–49.9	5.26–33.4				
Mg	420	320	1.27 ± 0.51	1.66 ± 0.74	0.30	0.40	0.40	0.52
			0.38–2.28	0.51–3.26				
Na	1500	1500	0.17 ± 0.11	0.11 ± 0.05	0.01	0.01	0.007	0.007
			0.05–0.45	0.04–0.20				
Mn^a^	2.3	1.8	0.24 ± 0.04	0.28 ± 0.14	10.4	13.3	12.2	15.5
			0.15–0.32	0.11–0.58				
Fe^b^	10	18	0.02 ± 0.005	0.01 ± 0.01	0.20	0.10	0.10	0.05
			0.01–0.03	<LOD-0.04				
P	700	700	7.25 ± 1.47	2.06 ± 0.64	1.00	1.00	0.29	0.29
			4.11–11.2	1.03–2.91				
Zn	11	8	0.02 ± 0.01	0.02 ± 0.005	0.18	0.16	0.18	0.25
			0.01–0.04	0.01–0.03				

^a^American recommendations [34]

^b^Polish recommendations [33]

LOD for Fe = 0.01 mg/100 g

In accordance with the EFSA [40] recommendations, tolerable daily intake (TDI) for Ni should not exceed 2.8 μg/kg for a person weighing 70 kg. Nickel levels in all analyzed tea infusions were <4.0 μg/100 g in Pu-erh (loose/bags) and fruit tea/black tea with fruits. It means that consumption of five cups of the analyzed tea do not exceed TDI (10.2 % of TDI), but can lead to its exceeding if tea intake is higher. Bioavailability of Ni from tea should be also taken into account, but there is no such data reported. Nickel deficiency has not been observed in humans, whereas exposure to its high levels may result in adverse health effects. High Ni concentration in tea can be due to the application of low-quality fertilizers contaminated with heavy metals that are deposited in soils and tea leaves [17].

## Conclusions

Nowadays, due to the increasing environment contamination, it is important to assess toxic metal and bioelement intake with food, including Pu-erh and fruit teas that consumption steadily increases. In our study, it was found that there is no health hazard associated with exposure to Cd and Pb via consumption of both kinds of the analyzed tea. However, if Ni contamination is observed, its TDI can be exceeded. Such situations can be possible in case of tea bag consumption, which as shown in statistical analysis are described mainly by heavy metals.

It was also concluded that multivariate techniques constitute efficient tools helpful in differentiation of tea samples in view of their quality, type, and confection. Magnesium, Ca, K, Na, P, Mn, Cu, Fe, Zn, Co, Cr, Ni, Cd, and Pb proved to be good descriptors for diversification of the analyzed samples.

## Abbreviations

CTC: (crush, tear, curl), rollers during the manufacturing of tea
CA: Cluster analysis
FA: Factor analysis
FAAS: Flame atomic absorption spectrometry
PTWI: Provisional tolerable weekly intake
RDA: Recommended dietary allowance
RSD: Relative standard deviation
SD: Standard deviation
